# Influence of stimuli emotional features and typicality on memory performance: insights from a virtual reality context

**DOI:** 10.1007/s00426-023-01850-8

**Published:** 2023-06-27

**Authors:** Irene Ceccato, Eleonora Ricci, Cristina Mazza, Emanuela Bartolini, Adolfo Di Crosta, Pasquale La Malva, Silvia Biondi, Marco Colasanti, Nicola Mammarella, Rocco Palumbo, Paolo Roma, Alberto Di Domenico

**Affiliations:** 1 Department of Neuroscience, Imaging and Clinical Sciences, University “G. d’Annunzio” of Chieti-Pescara, Chieti, Italy; 2 Department of Psychological, Health and Territorial Sciences, University “G. d’Annunzio” of Chieti-Pescara, Chieti, Italy; 3 Department of Human Neuroscience, Sapienza University of Rome, Rome, Italy

## Abstract

The present study tested the influence of stimuli emotional valence, emotional arousal, and typicality on memory recollection in three groups of participants exposed to the same environment through different modalities: in vivo exposure (i.e., real-life), 3D virtual reality (i.e., VR), and 2D pictures. Context-related free-recall, recognition accuracy, and recognition confidence were analyzed. The results showed that memory performance was best in the real-life modality, and participants in the VR and 2D pictures modalities performed comparably. Interesting effects of stimuli emotional valence and typicality emerged: in the VR and 2D pictures modalities, positive items were better recalled than negative items; typicality was relevant only in the real-life modality, with less common objects within the explored setting (i.e., an office) recalled more often. Furthermore, recognition accuracy and confidence were significantly higher in the real-life modality than in the VR and 2D pictures modalities. Further research is needed to support the creation of VR environments that are sufficiently comparable to real-life contexts in order to obtain higher ecological validity in studies of cognitive performance. In particular, the impact of stimuli typicality and emotional valence in VR contexts should be investigated to gain insight into how these features might improve memory recall in virtual scenarios.

**Supplementary Information:**

The online version contains supplementary material available at 10.1007/s00426-023-01850-8.

## Introduction

### Research comparing memory performance between real-life, VR, and 2D contexts

Memory describes the capacity to successfully encode, store, and retrieve the information that has been previously experienced (Zlotnik & Vansintjan, 2019). Memory is not a monolithic construct, but it includes different systems, such as sensory, short-term, and long-term memory. A further classification within the long-term memory system includes declarative (semantic, episodic, and autobiographical) and procedural memory (the reader interested in a detailed description of memory dimensions and mechanisms can refer to the extensive work of Baddeley et al. 2020). In the current study we focused on episodic memory and information retrieval. Episodic memory can be defined as the memory for specific events that occurred at a particular time and place (Anderson, 2020). Information retrieval is performed through the processes of recall and recognition. Recall refers to the ability to remember—and thereby reproduce—previously learned information. The simplest form of recall is free recall, which describes the process of recalling as many stimuli as possible, in no precise order, with no external cues. Recognition, on the other hand, refers to the ability to recognize that a particular stimulus or event (often presented within a set of distractors) has been previously experienced (Baddeley, 2004). Recognition performance, which is commonly measured in reference to signal detection theory (SDT; Green & Swets, 1966), is associated with recognition confidence (i.e., confidence in one’s ability to recognize stimuli). Consistently, recognition memory is based on two fundamental and different components: recollection and familiarity (see, for a review, Aggleton & Brown, 2006; Diana et al., 2007; Yonelinas, 2002). Recollection describes the retrieval of a specific, previously experienced stimulus, while familiarity represents a more global measurement of memory strength and confidence with respect to a specific event or stimulus (Yonelinas et al., 2010).

As demonstrated in the voluminous literature, memory performance, or the ability to correctly recall or recognize previously encoded information, is also intrinsically context-related (Smith, 1979; Smith & Vela, 2001), as retrieval is enhanced when the information is recalled in the same context as that in which it was encoded (see, e.g., Godden & Baddeley, 1975). However, it remains controversial whether memory performance in virtual reality (VR) environments is similar to memory performance in real life—and, if so, to what extent (Smith, 2019; van Helvoort et al., 2020). For instance, a recent study compared physiological features related to memory retrieval in real-life, VR, and conventional experimental conditions. Participants were shown the same event of a car driving in 2D video format, in 3D VR video, or as a physical enactment. No significant differences were detected in the recognition memory task between the real-life and VR conditions, but significant differences emerged between these conditions and the 2D video condition (Schöne et al., 2017), suggesting that VR and real-life contexts may be somewhat comparable and both better than 2D condition. Conversely, a study by Mania and Chalmers (2001) investigating memory recall in four experimental learning conditions (i.e., in-person, on a 3D desktop, with a 3D head-mounted display [HMD], via audio) found significantly superior recall in the in-person condition, compared to the VR condition. Likewise, Flannery and Walles (2003) compared item recognition for objects between participants in a virtual versus a real-life office. Scores were significantly greater for the real-life condition, suggesting non-comparable performance between real and virtual learning.

A parallel body of research has explored differences in memory performance between 2D and VR learning environments. Krokos et al. (2019) investigated memory in participants using virtual memory palaces (i.e., method of loci) presented through an HMD and participants using a traditional 2D desktop display. Participants in the HMD condition demonstrated higher memory recall compared to participants in the 2D desktop condition. However, other research comparing VR and 2D environments has produced contrasting results. For instance, Barreda-Ángeles et al. (2021) conducted a within-subjects study in which participants were asked to watch eight journalistic pieces on a 360°-video and on a 2D screen. After the video presentation, participants completed a recognition task and, a week later, free recall and cued recall tests. The authors found that the VR and immersive condition elicited lower recognition and cued recall of information, while no significant differences were found in the free recall task. Similarly, a study by Kisker et al. (2021b) exposed participants to a video in a PC condition and a VR condition, followed by an unannounced recognition memory test. The results showed that both groups performed equally well on the unannounced recognition memory test.

### The impact of stimuli’s emotional valence, emotional arousal, and typicality on items retrieval

The impact of coding context on memory performance may be partially influenced by the specific features of the memory stimulus, including its emotional valence and typicality. In fact, research has shown that stimuli emotional features (i.e., valence, arousal) have a significant impact on memory (for a review, see Kensinger & Schacter, 2016). Emotional valence concerns an individual’s definition of a stimulus as attractive or aversive (i.e., positive or negative, respectively). Several studies conducted in laboratory settings have shown that both positive and negative emotional stimuli are remembered better and more vividly than non-emotional stimuli (i.e., neutral stimuli; Kensinger, 2004; Kensinger & Corkin, 2004; Palumbo et al., 2023). Concerning arousal, which reflects an individual’s level of energy in response to a stimulus, research has shown that emotionally arousing items carry greater weight in cognitive processes and are better recalled than neutral stimuli (e.g., Kensinger & Corkin, 2003).

To date, few studies have evaluated differences in memory performance related to coding context (i.e., real-life, 2D, VR), even examining the influence of stimuli emotional valence and arousal. For example, it has been demonstrated that VR movies produce greater emotional arousal than 2D movies (Tian & Zhang, 2021).

With respect to typicality, previous studies have shown that stimuli that are easily distinguished from (and thus not in alignment with) their context are significantly better recalled (Bylinskii et al., 2015; Vogt & Magnussen, 2007; Watier & Collin, 2012). Mourkoussis et al. (2010) created two versions of a virtual academic office: one with highly detailed objects and another with only plain objects. Furthermore, items placed in the virtual office were either consistent (e.g., books, chair) or inconsistent with the environment (e.g., snake, dart board). Participants explored the virtual office using an HMD and were subsequently administered an old/new item recognition test. The results revealed no main effect concerning visual detail, but significantly better memory for the inconsistent items in the highly detailed condition, and no influence of visual detail on memory for the consistent items.

### Premises, aims, and hypotheses of the present research

Overall, the inconsistency encountered in the literature about memory performance is likely due to the wide variety of devices classified as VR. For example, Smith has illustrated three main subtypes of VR systems: Desktop-VR, Headset-VR (i.e., Head-Mounted Display, HMD), and Simulator-VR, each with different technical features (for a detailed description, see the review of Smith, 2019). Consequently, mixed results may depend on VR systems’ properties, such as visual fidelity and visual detail of the Virtual Environment (VE), and the elicited sense of presence (i.e., the feeling of “being there” subjects experience in VR) and immersiveness, which is determined by objective characteristics of the VR system. Also differentiating the studies is the methodology employed, which may include: (a) active vs. passive viewing; (b) interaction with the VE; (c) multimodal sensory stimuli. Furthermore, recent studies moved the focus of the study on memory processes, rather than performance, exploring electrophysiological correlates in the VR setting (Johnsdorf et al., 2023). Hence, a plain comparison between studies is difficult.

Given the contradictory results reported in the literature, the first aim of the present study was to gain insight into the impact of coding context on memory performance. Specifically, three groups of young adults were exposed to the same environment (i.e., an academic office) in three different coding contexts: (a) a real office; (b) a panorama 360° photo of the office loaded inside a Head-Mounted Display (HMD, i.e., Meta Quest 2); and (c) 2D pictures of the office. In doing that, we considered different facets of memory performance and investigated both free recall and recognition tasks. Although the literature is inconsistent, we hypothesize that the memory performance of participants exposed to a real-life context would be comparable to that of the subjects in VR; conversely, the 2D condition would lead to worse performance. Furthermore, as recognition is generally easier than recall with no external cues (Baddeley et al., 2020; Rhodes et al., 2019), we hypothesized that potential differences between coding contexts would be magnified in the free recall task compared to the recognition task.

The second aim of the study was to examine the impact of stimuli features (emotional valence, emotional arousal, and typicality) on item retrieval, comparing within the same study three experimental coding conditions (i.e., real-life, 2D, and HMD-VR). Finally, we tested recognition confidence to evaluate whether the coding contexts may differently affect the subjective assessment of memory performance, possibly revealing a discrepancy with the objective performance. To the best of our knowledge, few studies (e.g., Mania et al., 2003) have studied recognition confidence related to VR environments. Findings showed that participants in the real-life condition achieved the most accurate and confident recognition performance, followed by participants in the VR condition, and, lastly, participants in the 2D condition, who showed the lowest accuracy and confidence recognition.

## Materials and methods

### Participants

One hundred and twenty-three young adults were recruited for and voluntarily participated in the study. The inclusion criteria were: (i) aged 18 years and older and (ii) able to comprehend the Italian language perfectly. Four participants (3.25%) were excluded due to an invalid procedure, caused by problems associated with the VR equipment (i.e., HMD-VR Meta Quest 2). Thus, the final sample was comprised of 119 participants (see Table 1): 62 male (52.1%) and 57 female (47.9%), aged 18–35 years (*M* = 24.20, *SD* = 4.13). Most participants were Italian citizens (*n* = 118, 99.2%) and living in central Italy (*n* = 107, 89.9%). They were predominantly students (*n* = 77, 64.7%) whose highest educational achievement was high school (*n* = 67, 56.3%). The majority had no visual impairment (51.4%). Sensitivity analyses were conducted using G*power (version 3.1.9.7), to compute the minimum effect size that could be detected given the alpha, power, and sample size (Perugini et al., 2018). The results for a one-way ANCOVA involving three groups with alpha = 0.05, power = 0.80, and 119 participants indicated sufficient power to detect a medium effect size, partial η^2^ ≥ 0.08. The results for a repeated measure within-between interaction ANOVA involving three groups and two measures as within-subject factors, given alpha = 0.05, power = 0.80, and 119 participants, indicated sufficient power to detect a medium effect size, partial η^2^ ≥ 0.02.

**Table 1:** Descriptive statistics of the sample

	Real-life	HMD-VR	2D pictures	Total
	*n* (%)	*n* (%)	*n* (%)	*N* (%)
Biological sex				
Female	22 (55.0)	18 (45.0)	17 (43.6)	57 (47.9)
Male	18 (45.0)	22 (55.0)	22 (56.4)	62 (52.1)
Citizenship				
Italian	39 (97.5)	40 (100)	39 (100)	118 (99.2)
Other	1 (2.5)	0 (0)	0 (0)	1 (0.8)
Region				
North	0 (0)	2 (5)	1 (2.6)	3 (2.5)
Central	36 (90)	36 (90)	35 (89.7)	107 (89.9)
South	4 (10)	2 (5)	3 (7.7)	9 (7.6)
Educational level				
Middle school diploma	1 (2.5)	0 (0)	0 (0)	1 (0.8)
High school diploma	11 (27.5)	27 (67.5)	29 (74.4)	67 (56.3)
Degree	24 (60)	13 (32.5)	6 (15.4)	43 (36.1)
Post-graduate	4 (10)	0 (0)	4 (10.3)	8 (6.7)
Occupation				
Unemployed	1 (2.5)	1 (2.5)	2 (5.1)	4 (3.4)
Student	17 (42.5)	30 (75)	30(76.9)	77 (64.7)
Employee	18 (45)	6 (15)	3 (7.7)	27 (22.7)
Freelancer	4 (10)	3 (7.5)	4 (10.3)	11 (9.2)
Vision impairment				
No	19 (47.5)	20 (50)	22 (56.4)	61 (51.3)
Yes	21 (52.5)	20 (50)	17 (43.6)	58 (48.7)
Use of glasses/contact lenses				
No	23 (57.5)	21 (52.5)	22 (56.4)	66 (55.5)
Yes	17 (42.5)	19 (47.5)	17 (43.6)	53 (44.5)
Prior experience of VR				
No	30 (75)	26 (65)	26 (66.7)	82 (68.9)
Yes	10 (25)	14 (35)	13 (33.3)	37 (31.1)

Participants were randomly allocated to one of three experimental conditions according to a manipulated variable (i.e., the coding environment; see the “Experimental procedure” section for a detailed description):

- *Real-life* (*M*_age_ = 26.53, *SD* = 3.60): group 1 was composed of 40 participants who visited the academic office in vivo.
- *HMD-VR* (*M*_age_ = 23.25, *SD* = 4.19): group 2 was composed of 40 participants who visited the academic office in 3D VR, using a Meta Quest 2.
- *2D pictures* (*M*_age_ = 22.79, *SD* = 3.60): group 3 was composed of 39 participants who observed 2D pictures of the academic office on a computer.

### Measures

A socio-demographic questionnaire was administered to all participants to gather personal information about biological sex, age, education, occupational status, region of residence, citizenship, and medical diagnoses. Data concerning visual impairment and prior experience of VR were also retrieved.

The experimental memory tasks were the following:

#### Free recall task

Participants were given 4 min to write down all of the objects in the room they visually explored. Globally, 53 objects were identified across all groups. The free recall total score was calculated as a ratio of the number of objects recalled to the total number of recallable objects (i.e.,* N* = 53). In a subsequent phase, a rating study was conducted online on an independent sample of young adults (*N* = 138)—not undergoing the memory assessment—to evaluate each object according to three dimensions: valence (i.e., from negative/low to positive/high), arousal (i.e., from low to high), and typicality (i.e., commonality of the object in an office setting; from low to high). More details on the rating study are provided in the “Supplementary Information”.

Based on the average ratings, each object was categorized as either low or high on each dimension. Of note, the three dimensions were treated independently. For example, a dirty water bottle was classified as high on the arousal dimension but low on the valence and typicality dimensions. Subsequently, six free recall subscores were calculated: two (i.e., low and high) for each dimension (i.e., valence, arousal, typicality). Subscores were calculated as a ratio of the number of objects recalled to the total number of objects in a given category. For example, the high typicality category comprised 34 objects (out of 53). If a participant recalled eight objects in this category, they obtained a high typicality subscore of 0.23. The analyses of these three dimensions provided insight into the impact of coding context (i.e., real-life, HMD-VR, 2D pictures) on memory performance, considering stimulus emotional valence and typicality.

#### Recognition task

**Fig. 1:**
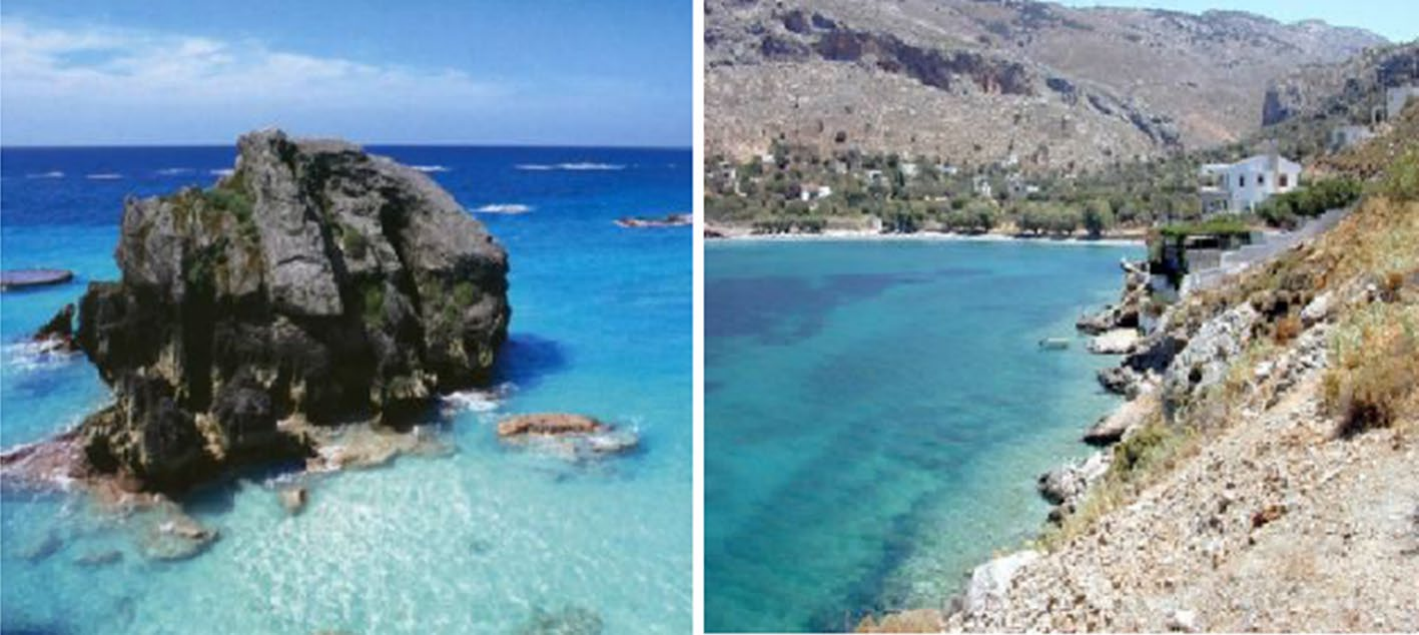
Two Items from the Visual Recognition Task. The image on the left was a painting that hung on the wall of the visually explored room, while the image on the right was not present in the room

Participants were shown 20 pictures of objects on a 27″ personal computer, with no time limit. Ten of these pictures depicted objects that were present in the room the participants visually explored (i.e., “old items”). The remaining 10 pictures depicted objects that were not present in the room (i.e., “new items”). Figure 1 presents two pictures included in the visual recognition task. Note that new items were not always perfectly paired with old items. Participants saw one picture at a time and were instructed to indicate if the object depicted in the picture was present in the room they visually explored, by selecting one of five options: 1 (*surely I did not see it in the room*), 2 (*maybe I did not see it in the room*), 3 (*I do not know if I saw it in the room*), 4 (*maybe I saw it in the room*), and 5 (*surely I saw it in the room*). This response scale was designed to analyze both accuracy (i.e., according to SDT; Stanislaw & Todorov, 1999) and recognition confidence.

In line with the SDT framework, recognition performance was explored through the calculation of several variables: First, the number of hits (i.e., number of correctly recognized old items) and the complementary number of misses (i.e., number of unrecognized old items); for example, out of 10 old items, a participant with a hit score of 7 had a miss score of 3. Second, the number of false alarms (i.e., number of misrecognized new items) and the complementary number of correct rejections (i.e., number of correctly identified new items). Third, a discriminability parameter (*d*′), reflecting the extent to which two “things” (conventionally labeled “signal” and “noise”) could be distinguished. In the recognition task, old items were the “signal” to be detected, while new items were the “noise” to be ignored. The discriminability parameter *d*′ was thus calculated as the difference between the *z*-transformed scores of the distributions of hits and false alarms, ranging from 0 (i.e., no discrimination) to infinity (i.e., perfect discrimination). And fourth, a decision bias parameter I, indexing the propensity to label an object as old (or new). The C parameter was calculated as the sum of the *z*-transformed scores of the distributions of hits and false alarms, divided by 2 and multiplied by − 1. C = 0 reflected a “neutral” response tendency, with no decision bias towards either response type; C < 0 indicated a bias towards “old” responses (resulting in more hits, as well as more false alarms); and C > 0 indicated a preference for “new” responses (resulting in fewer hits and fewer false alarms). For old items, responses 4 and 5 were coded as correct recognition (i.e., hits), while responses 1, 2, and 3 were coded as misses. For new items, responses 1 and 2 were coded as correct rejection, while responses 3, 4, and 5 were coded as false alarms.

Subsequently, recognition confidence for the correct identification as old (i.e., hit) or new (i.e., correct rejection), as well as for false alarms and misses, was calculated by recoding each answer, as described in Table 2. Average scores were then computed. Hit confidence and correct rejection confidence ranged from 0 to 2, while false alarm confidence and miss confidence ranged from 1 to 2.

**Table 2:** Coding criteria for confidence in the recognition task

		Response				
		1 Surely new	2 Maybe new	3 Do not know	4 Maybe old	5 Surely old
Item	Old	Miss confidence = 2	Miss confidence = 1	Hit confidence = 0	Hit confidence = 1	Hit confidence = 2
	New	Correct rejection confidence = 2	Correct rejection confidence = 1	Correct rejection confidence = 0	False alarm confidence = 1	False alarm confidence = 2

#### Sense of presence in the VR environment

Two individual items were employed to measure the sense of presence, with the goal of ensuring that the VR environment had elicited the experience of "being there" in participants. Specifically, two questions were asked: the first (i.e., “*I felt completely immersed*”) was adapted from Jennett et al.’s (2008) scale and assessed on a 7-point Likert scale ranging from 1 (*not at all*) to 7 (*very strongly*), as also applied in previous research (e.g., Hudson et al., 2019). The second question (i.e., “*I felt like I was inside the room*”) was adapted from Wagler and Hanus’s (2018) scale of spatial presence and was assessed on a 7-point Likert scale ranging from 1 (*not at all*) to 7 (*a lot*).

Furthermore, to control for baseline memory performance the following measures were administered:

#### Rivermead behavioural memory test

The Rivermead Behavioural Memory Test (RBMT-III; Wilson et al., 1985; Italian validation: Beschin et al., 2013) is an ecological instrument that evaluates respondents’ capacity to use memory in ordinary circumstances. The RBMT is composed of 14 subtests, which assess visual memory, verbal memory, and recall memory, both immediate and delayed. In the present study, only the Figure Recognition subtest was used to assess participants’ ability to recall previously displayed images from a larger set. This subtest consists of 15 drawings of ordinary objects that are shown sequentially, each for 3 s. In part 1 of the subtest, respondents are asked to name the object represented in the 15 drawings; in part 2, measuring deferred recognition, they are instructed to identify the original objects from a group of 30 drawings (i.e., a set that includes 15 distractor drawings). If respondents make a mistake in their naming of an object, the examiner corrects them and notes the potential presence of language or perceptual problems. The RBMT has been shown to have good ecological validity and to successfully predict real-life behavior and deficits outside the evaluation situation. The present study considered the number of images correctly recognized (ranging from 0 to 15).

#### Corsi block-tapping test

The Corsi block-tapping test (CBT; De Renzi & Nichelli, 1975; Spinnler & Tognoni, 1987) measures the quantity of information that can be held in visuo-spatial memory (i.e., short-term memory). The stimulus consists of a wood board (32 × 25 cm) upon which nine progressively numbered cubes (4.5 × 4.5 × 4.5 cm) are asymmetrically placed. Each cube displays a number, which faces the examiner (who sits across from the participant). The examiner demonstrates a sequence of numbers by tapping on the cubes at a rate of 2 s per cube. Subsequently, the respondent must replicate the sequence by touching the cubes in the same order. This process is repeated, with the sequence length increasing from a minimum of 3 to a maximum of 10. For every sequence length, there are two sequences performed. When the participant correctly reproduces a sequence, the examiner moves on to the next longest sequence length. The participant’s visuo-spatial memory span corresponds to the longest sequence of numbers reproduced correctly.

### Experimental procedure

The present study comprised part of a wider research project. The experimental procedure was conducted in the Department of Human Neuroscience laboratories at the “Sapienza” University of Rome, in October 2021. It was divided into three stages, each lasting approximately 30 min. The study was approved by the local ethics committee (Board of the Department of Human Neuroscience, Faculty of Medicine and Dentistry, Sapienza University of Rome—n° 15/2021), according to the Declaration of Helsinki.

#### Stage 1

After arriving at the laboratory, participants provided written informed consent, filled out the socio-demographic questionnaire, and completed the RBMT-III Figure Recognition subtest and Corsi block-tapping text (see the “Measures” section).

#### Stage 2

Participants were randomly allocated to one of three experimental conditions. All groups were asked to observe an academic office and memorize as many objects as possible over a period of 2 min. However, the three experimental conditions were differentiated according to the coding environment in which the procedure took place (i.e., real-life, HMD-VR, 2D pictures).

- *Real-life group*: Participants were positioned in the middle of the academic office—a room within the university—and instructed to remain stationary and only rotate to observe the room and the objects within it. Specifically, the instructions were as follows: *“We will now take you into a room. You must observe the room carefully for 2 min, memorizing as many objects as possible. You must remain in the spot in which we position you. However, you can turn your body in all directions”.*
- *HMD-VR group*: Participants were taken to a neutral room and instructed to put on a Head-Mounted Display (HMD; i.e., Meta Quest 2^1^), which displayed a panorama 360° picture of the academic office, taken by a professional photographer with a Lapbano Pilot One EE. The 360° picture was taken from the same point where participants in the Real-life group were standing. Participants were instructed on the use of the Meta Quest 2 (e.g., not moving outside the planned area, physically turning on themselves to see all parts of the room) and asked to observe the academic office and memorize as many details as possible over a period of 2 min. Specifically, the instructions were as follows: *“Now you are going to participate in a virtual reality experience, wearing this visor. You will enter a room and explore it in 360 degrees. Observe the room carefully for 2 min, memorizing as many objects as possible. You must remain still, with the exception that you can turn your head and rotate your body in all directions.”*

Following this step, participants’ sense of presence was assessed through two questions (see the “Sense of presence” paragraph).

**Fig. 2:**
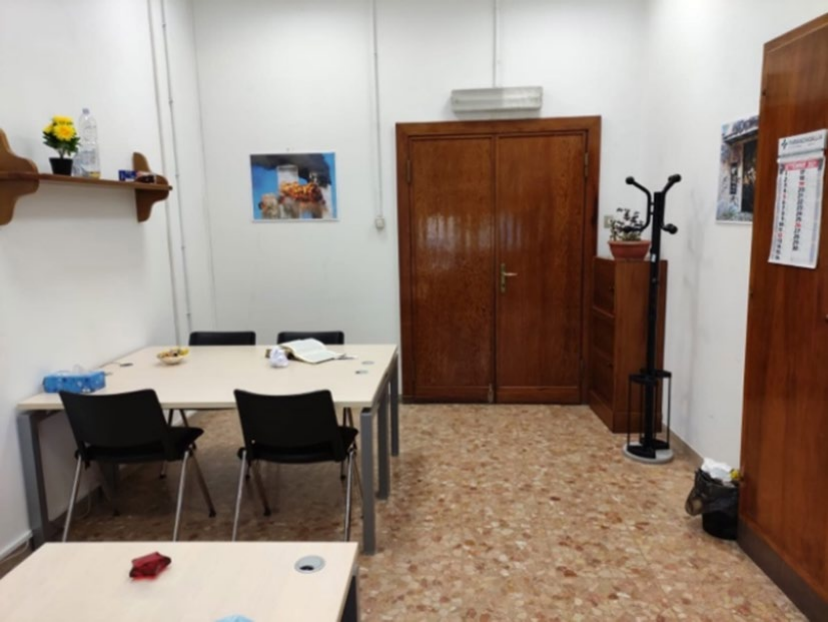
Sample picture used in the 2D condition

- *2D pictures group*: Participants were taken to a neutral room, where they were seated in front of a 27″ computer monitor upon which eight 2D pictures (2048 × 1537-pixel, 3 megapixels) displayed the same academic office from the same point of view of participants in the Real-life and HMD-VR conditions—in Fig. 2 is reported one of the pictures used in the 2D condition (see Supplementary Information for the remaining). Participants could scroll back and forward across the pictures as they wanted, and they were told that they had 2 min to look at and memorize as many objects as possible in the academic office. Specifically, the instructions were as follows: “*Now we will show you some pictures of a room. Observe the room carefully for 2 min, memorizing as many objects as possible*.”

We loaded a panorama 360° photo into HMD Meta Quest 2 to avoid using a computer-generated scenario, representing an academic office in an artificial way, to make the VR stimulus as similar to reality as possible. Additionally, in the 2D condition was not shown the panorama 360° picture used in the HMD-VR condition as its resolution did not allow to see clearly all the objects in the office. Indeed, this picture was developed for a VR device and thus was unadaptable for a computer monitor.

#### Stage 3

Participants in all groups returned to the original reception room, where they completed the two experimental tasks, starting with the free recall task. Subsequently, they completed the recognition task (see the “Measures” section) on a 27″ personal computer, with no time limit.

## Statistical analysis

One-way ANOVAs and chi-square tests, depending on the nature of the variable, were run to test group differences in socio-demographic features and general memory performance (i.e., scores on the RBMT and CBT). A single sample *t*-test was run to test the sense of presence. Zero-order correlations were computed between socio-demographic variables, general memory performance, free recall, and recognition. A univariate ANCOVA using group (i.e., real-life, HMD-VR, 2D pictures) as an independent factor was performed on the free recall total score. Subsequently, three repeated-measure ANCOVAs on the three recall subscores (i.e., valence, arousal, typicality) were run, entering the low versus high level of each dimension as a within-subject factor and group as a between-subject factor. Finally, univariate ANCOVAs were computed considering group (i.e., real-life, HMD-VR, 2D pictures) as an independent factor and SDT recognition parameters and recognition confidence (i.e., number of hits, number of false alarms, *d*′, C, hit confidence, correct rejection confidence, miss confidence, false alarm confidence) as dependent variables. Age and education were entered as covariates. All post hoc analyses were Bonferroni adjusted. All analyses were performed using SPSS version 27 software.

## Results

### Preliminary analyses

Analyses were run to determine whether the three experimental groups were comparable in terms of socio-demographic features and memory performance (see Table 3). No differences in gender distribution or general memory performance emerged between groups. Scores on the RBMT Figure Recognition subtest and CBT for the three groups corresponded to general population averages (Beschin et al., 2013; Spinnler & Tognoni, 1987). However, significant differences in age and education were found, with participants in the real-life group older and more educated than participants in the other conditions. Hence, subsequent analyses controlled for age and education.^2^ Table 4 presents the zero-order correlations between all of the considered variables.

**Table 3:** Descriptive information for the three experimental groups and results of the group comparison

	Real-life (*n* = 40)	HMD-VR (*n* = 40)	2D pictures (*n* = 39)	Group comparison	Post hoc
Age	26.53 (3.60)	23.25 (4.19)	22.79 (3.60)	***F*(2,116) = 11.34** ***p* < 0.001** **Partial η**^**2**^** = 0.16**	Real-life > HMD-VR, 2D pictures HMD-VR = 2D pictures
Gender	M = 55% F = 45%	M = 45% F = 55%	M = 44% F = 56%	χ^2^(2) = 1.23 *p* = 0.540	–
Education	3.78 (0.66)	3.33 (0.47)	3.36 (0.67)	***F*(2,116) = 6.80** ***p* = 0.002** **Partial η**^**2**^** = 0.10**	Real-life > HMD-VR, 2D pictures HMD-VR = 2D pictures
RBMT	14.73 (0.64)	14.60 (0.67)	14.74 (0.55)	*F*(2,116) = 0.62 *p* = 0.537 Partial η^2^ = 0.01	–
CBT	5.98 (1.35)	6.28 (1.47)	5.92 (1.42)	*F*(2,116) = 0.72 *p* = 0.490 Partial η^2^ = 0.01	–

*RBMT* Rivermead Behavioural Memory Test—Figure Recognition subtest, *CBT* Corsi Block-Tapping test of visuo-spatial memory span. Means (and standard deviations) are reported for all continuous variables. For gender, frequencies (in %) are reported. Post hoc analyses were Bonferroni adjusted. Statistically significant effects (*p* < 0.05) are in bold.

**Table 4:** Correlations between socio-demographic characteristics, memory performances, free recall, and recognition

	Gender	Education	RBMT	CBT	FRT	Hit	False alarm	*d′*	C	Hit Conf	CR Conf	FA Conf	Miss Conf
Age	− 0.01	0.58***	0.10	− 0.14	0.38***	0.27**	− 0.10	0.29**	− 0.16^+^	0.26**	0.17^+^	0.21*	0.08
Gender		− 0.17^+^	− 0.07	0.10	− 0.24**	− 0.01	0.03	− 0.01	− 0.03	0.01	− 0.05	− 0.21*	0.01
Education			0.09	− 0.09	0.42***	0.23*	− 0.01	0.19*	− 0.17^+^	0.15	0.16^+^	0.15	0.03
RBMT				0.04	0.13	0.05	− 0.04	0.07	− 0.01	0.02	0.13	0.17^+^	0.12
CBT					− 0.01	0.04	− 0.07	0.07	− 0.01	− 0.11	− 0.13	− 0.09	− 0.10
FRT						0.39***	− 0.01	0.33***	− 0.29**	0.38***	0.25**	0.42***	0.19*
Hit							− 0.05	0.77***	− 0.79***	0.55***	0.13	0.22*	0.13
False alarm								− 0.59***	− 0.64***	0.05	0.05	− 0.19*	− 0.11
*d′*									− 0.24**	0.40***	0.09	0.32***	0.17^+^
C										− 0.45***	− 0.15	− 0.06	− 0.05
Hit Conf											0.40***	0.52***	0.30***
CR Conf												0.24**	0.71***
FA Conf													0.21*

*RBMT* Rivermead Behavioural Memory Test, *CBT* Corsi block-tapping test of visuo-spatial memory span, *FRT* free recall total score, *Hit Conf* hit confidence, *CR conf* correct rejection confidence, *FA conf* false alarm confidence, *Miss conf* miss confidence

Pearson’s *r* is reported for all variables but gender. Point-biserial correlations are reported for the associations between gender and other variables

^+^*p* < 0.10; *p < 0.05; **p < 0.01; ***p < 0.001

#### Sense of presence

Results revealed that participants in the HMD-VR condition experienced an appropriate sense of presence in response to both questions (“*I felt completely immersed*”: *M* = 5.48; *SD* = 1.43; “*I felt like I was inside the room*”: *M* = 5.52; *SD* = 1.71). A single sample *t*-test showed that the average score of both questions significantly differed from the central value of 4 (first question: *t*_30_ = 5.759, *p* < 0.001; second question: *t*_30_ = 4.936, *p* < 0.001).

### Group differences in free recall

**Fig. 3:**
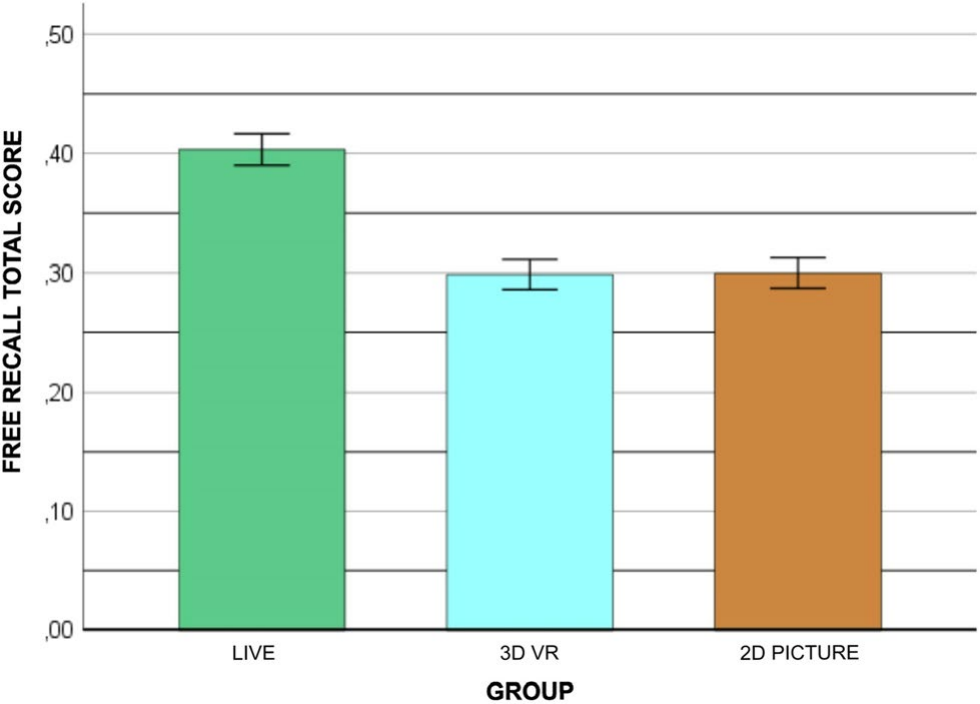
Free recall performance in each experimental group. Error bars represent ± 1 *SE*. Covariates were evaluated at the following values: age = 24.20; education = 3.49

A univariate ANCOVA with group as the independent factor (three levels: real-life, HMD-VR, 2D pictures) was performed on the free recall total score. Age and education were entered as covariates. The results revealed a main effect of group, *F*(2,114) = 19.44, *p* < 0.001, partial η^2^ = 0.25. Post hoc analyses showed that the real-life group outperformed both the HMD-VR and the 2D picture groups (Fig. 3). Education was a significant covariate, *F*(1,114) = 7.44, *p* = 0.007, partial η^2^ = 0.06, but not age, *F*(1,114) = 0.16, *p* = 0.688, partial η^2^ = 0.001.

### Effects of valence, arousal, and typicality on free recall

Analyses aimed at examining the role of object characteristics on recall, in terms of the three dimensions of valence, arousal, and typicality. Three repeated measure ANCOVAs were run on recall scores, entering dimension level (i.e., low, high) as a within-subject factor and group (i.e., real-life, HMD-VR, 2D pictures) as a between-subject factor.

**Fig. 4:**
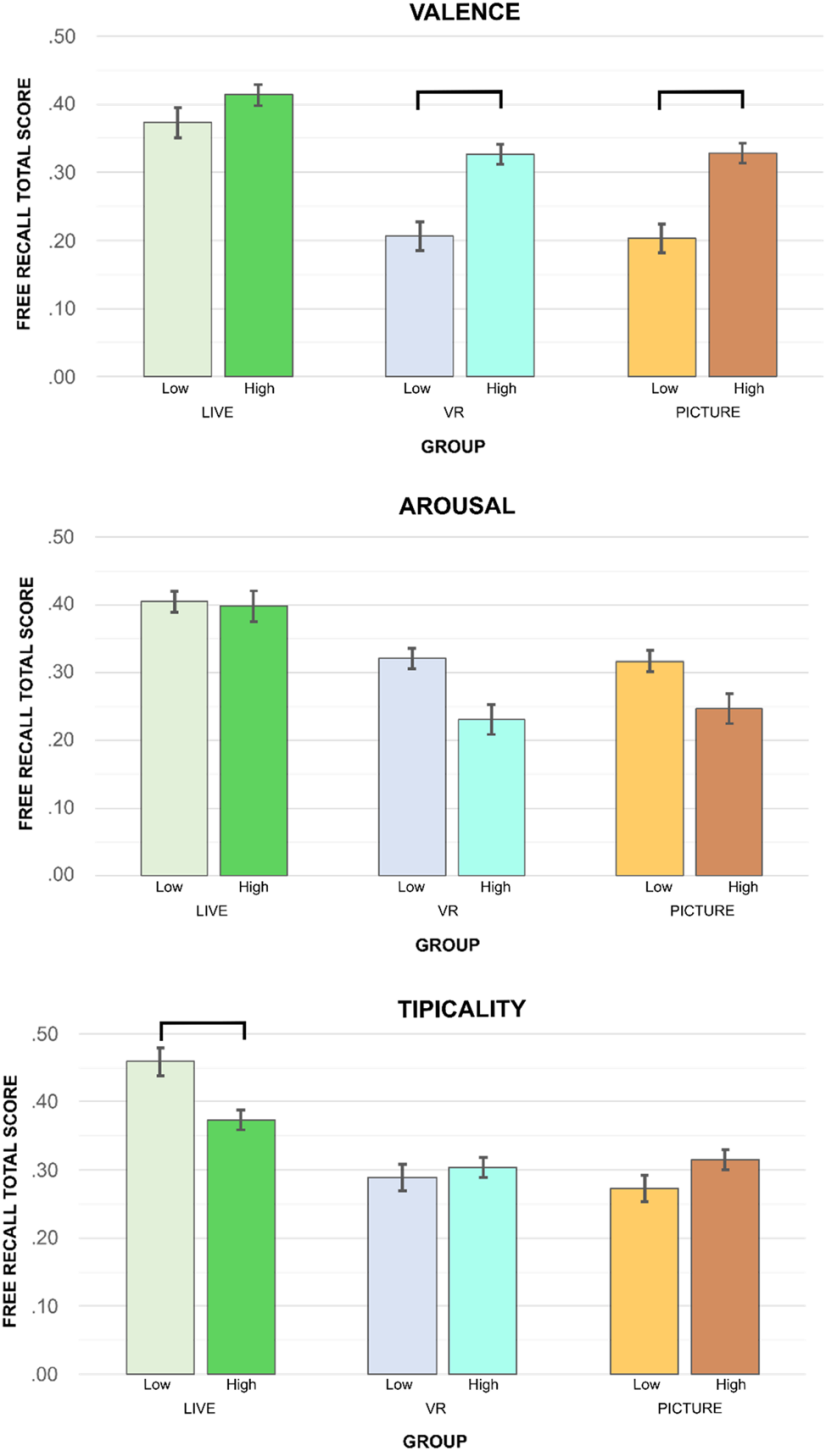
Free recall performance (valence, arousal, and typicality dimensions) as a function of Group (Real-life, HMD-VR, 2D Pictures) and Level (Low, High). Error bars represent ± 1 *SE*. Squared brackets indicate statistically significant differences (*p* < 0.001). Covariates were evaluated at the following values: age = 24.20; education = 3.49

For valence, the main effect of the experimental group was significant, *F*(2,114) = 26.56, *p* < 0.001, partial η^2^ = 0.32, with participants in the real-life group outperforming the other two experimental groups. The main effect of level was not significant, *F*(1,114) = 2.49, *p* = 0.117, partial η^2^ = 0.02. Crucially, a significant interaction effect was found between group and level, *F*(2,114) = 3.09, *p* = 0.049, partial η^2^ = 0.05. Post hoc analyses (Bonferroni adjusted) indicated that, in the real-life condition, there were no differences in the recall of low valence (*M* = 0.39, *SD* = 0.17) versus high valence objects (*M* = 0.42, *SD* = 0.10, *p* = 0.131). In contrast, in both the HMD-VR and the 2D picture conditions, recall was higher for objects with high valence (HMD-VR: *M* = 0.32, *SD* = 0.09; 2D pictures: *M* = 0.32, *SD* = 0.09) than for objects with low valence (HMD-VR: *M* = 0.20, *SD* = 0.10; 2D pictures: *M* = 0.19, *SD* = 0.11), *p*s < 0.001 (Fig. 4). Education was a significant covariate, *F*(1,114) = 3.98, *p* = 0.048, partial η^2^ = 0.03, while age was not, *F*(1,114) = 1.59, *p* = 0.210, partial η^2^ = 0.01.

For arousal, only the main effect of the experimental group emerged, *F*(2,114) = 23.59, *p* < 0.001, partial η^2^ = 0.29, indicating the superiority of the real-life condition in recall performance. Level had no main, *F*(1,114) = 2.10, *p* = 0.150, partial η^2^ = 0.02, or interaction, *F*(2,114) = 2.26, *p* = 0.109, partial η^2^ = 0.04, effects on recall. Education was a significant covariate, *F*(1,114) = 4.04, *p* = 0.047, partial η^2^ = 0.03, while age was not, *F*(1,114) = 1.69, *p* = 0.196, partial η^2^ = 0.01.

For typicality, a significant main effect of the experimental group emerged, *F*(2,114) = 23.99, *p* < 0.001, partial η^2^ = 0.30, confirming the real-life group’s superiority, while level had no main effect on recall, *F*(1,114) = 2.46, *p* = 0.120, partial η^2^ = 0.02. Of interest, a significant interaction effect between group and level was found, *F*(2,114) = 8.25, *p* < 0.001, partial η^2^ = 0.13. Post hoc analyses showed that, in the real-life condition, low typicality objects (*M* = 0.48, *SD* = 0.12) were recalled more frequently than high typicality objects (*M* = 0.38, *SD* = 0.10), *p* < 0.001. In contrast, in the HMD-VR condition, no difference (*p* = 0.513) between low (*M* = 0.28, *SD* = 0.12) and high typicality (*M* = 0.30, *SD* = 0.09) objects emerged. In the 2D picture condition, the difference between low and high typicality objects approached statistical significance (*p* = 0.053) (low: *M* = 0.26, *SD* = 0.12; high: *M* = 0.31, *SD* = 0.08), in the direction of a better recall of high typicality objects. Education was a significant covariate, *F*(1,114) = 5.65, *p* = 0.019, partial η^2^ = 0.05, while age was not, *F*(1,114) = 0.94, *p* = 0.334, partial η^2^ = 0.01.

### Group differences in recognition

A series of univariate ANCOVAs using the experimental group (i.e., real-life, HMD-VR, 2D pictures) as the independent factor was performed on the four SDT parameters (i.e., number of hits, number of false alarms, *d′*, C). Age and education were entered as covariates. Table 5 presents the means and standard deviations.

**Table 5:** Means (and standard deviations) for recognition

	Real-life *M *(*SD*)	HMD-VR *M *(*SD*)	2D pictures *M *(*SD*)
Number of hits	8.30 (1.14)	6.43 (1.95)	6.03 (1.72)
Number of false alarms	1.00 (1.68)	1.08 (1.05)	1.28 (1.10)
*d′*	2.28 (0.68)	1.67 (0.68)	1.44 (0.57)
C	0.14 (0.37)	0.41 (0.36)	0.44 (0.36)
Hit confidence	1.83 (0.15)	1.53 (0.23)	1.55 (0.26)
Correct rejection confidence	1.68 (0.22)	1.46 (0.31)	1.40 (0.30)
Miss confidence	1.76 (0.27)	1.59 (0.28)	1.55 (0.33)
False alarm confidence	1.92 (0.11)	1.64 (0.26)	1.73 (0.26)

For the number of hits, a main effect of the experimental group emerged, *F*(2,114) = 15.63, *p* < 0.001, partial η^2^ = 0.22. Specifically, post hoc analyses showed that the real-life group outperformed both the HMD-VR and the 2D picture groups. In contrast, group had no effect on the number of false alarms, *F*(2,114) = 0.27, *p* = 0.765, partial η^2^ = 0.005. A significant effect of group was found for *d′*, *F*(2,114) = 12.51, *p* < 0.001, partial η^2^ = 0.18, again showing that participants in the real-life group were more able to discriminate between new and old items than participants in the other two groups. Finally, for the decision bias C, a significant effect of group emerged, *F*(2,114) = 6.49, *p* = 0.002, partial η^2^ = 0.10. Post hoc analyses indicated that, although all groups showed a tendency to classify objects as new, this bias was significantly higher in the HMD-VR (*p* = 0.009) and 2D picture groups (*p* = 0.004), compared to the real-life group. Age and education were not significant covariates in any of the four models described, *F*s(1,114) ≤ 1.22, *p*s ≥ 0.272, partial η^2^ ≤ 0.01.

To explore recognition confidence, four ANCOVAs were conducted, one for each confidence score. Group was entered as the independent variable, while age and education were entered as covariates. In all four models, the main effect of the experimental group was significant (i.e., hit confidence: *F*(2,114) = 19.23, *p* < 0.001, partial η^2^ = 0.25; correct rejection confidence: *F*(2,113) = 8.57, *p* < 0.001, partial η^2^ = 0.13; miss confidence: *F*(2,112) = 5.15, *p* = 0.007, partial η^2^ = 0.08; false alarm confidence: *F*(2,113) = 13.22, *p* < 0.001, partial η^2^ = 0.19). Post hoc analyses showed that confidence was always higher in the real-life group compared to both the HMD-VR and the 2D picture groups, *p* ≤ 0.040.

Age and education were not significant covariates in any of the four models, *F*s(1,114) ≤ 0.99, *p*s ≥ 0.322, partial η^2^ ≤ 0.01.

For the number of hits and the number of false alarms, scores ranged from 0 to 10. For hit confidence and correct rejection confidence, scores ranged from 0 to 2. For miss confidence and false alarm confidence, scores ranged from 1 to 2.

## Discussion

The present study tested the impact of coding context on memory performance, as well as the influence of stimuli emotional valence, emotional arousal, and typicality on recollection. Three groups of participants were exposed to the same coding environment through different modalities: in vivo exposure (i.e., real-life), 3D virtual reality (i.e., Head-Mounted Display VR), and 2D pictures. Memory performance was assessed using both free recall and recognition tasks, while also considering recognition confidence. Overall, participants who performed the experimental task in the real-life encoding context showed higher memory performance in free recall, recognition, and recognition confidence than those who completed the task in the HMD-VR and 2D picture encoding contexts. In addition, memory performance in the HMD-VR and 2D image encoding contexts was comparable. Interestingly, emotional valence influenced the free recall of items in HMD-VR and 2D picture coding contexts, with positive items recalled more often. Finally, typicality influenced the free recall of items only in the real-life coding context: items less common within an academic office were recalled more often.

The first aim of the present study was to gain insight into the impact of coding context on memory performance. The findings underlined that free recall and recognition were significantly better for the real-life coding context, with participants remembering and recognizing more objects, on average, compared to participants in the HMD-VR and 2D picture conditions. These results align with the works of Mania and Chalmers (2001) and Flannery and Walles (2003), founding that memory recall and recognition performance, respectively, were significantly higher in an “in-person” coding condition compared to an HMD-VR coding condition. Of interest, in the present study, participants in the HMD-VR coding environment did not show enhanced memory performance compared to participants in the 2D picture coding condition; rather, memory performance between these conditions was similar. Hence, the present results are in line with previous findings showing no significant differences in recognition performance between participants exposed to 2D and VR coding environments (Barreda-Ángeles et al., 2021; Kisker et al., 2021b).

The second aim of the present research was to explore the influence of emotional valence, arousal, and typicality on memory performance. Interesting effects emerged regarding emotional valence and typicality. Specifically, emotional valence impacted the free recall of objects only in the HMD-VR and 2D picture contexts, whereby positive items were more accurately recalled than negative ones. These findings add to the large body of literature on the impact of emotional stimuli on memory performance (e.g., Fairfield et al., 2017; Kensinger & Schacter, 2016; Mammarella et al., 2016; Penolazzi et al., 2010). Moreover, objects connoted by a positive valence tended to be better recalled by participants in both the HMD-VR and the 2D picture contexts, compared to participants in the real-life context. This suggests that the emotional connotation of stimuli may play a differential role in memory performance, depending on the type of learning environment. However, the influence of stimuli emotional valence on memory performance in VR settings requires further investigation. Indeed, some research suggested that perceived emotional valence may change depending on the coding context, with the same stimulus eliciting even opposite emotions between VR and 2D conditions (Schöne et al., 2021).

Conversely, the present results highlight that typicality was relevant only in the real-life coding context, in which objects that were less commonly found within an office were recalled more often. These findings align with the results of previous research showing that stimuli that stand out from (and are thus not aligned with) their context are significantly better recalled (Bylinskii et al., 2015; Vogt & Magnussen, 2007; Watier & Collin, 2012). However, in the present study, typicality did not influence memory performance in the VR condition. This contradicts the findings of Mourkoussis et al. (2010), which showed better recall for non-typical items in a VR scenario. It could be argued that the degree of visual detail is superior in real-life contexts, relative to VR and 2D picture environments. Thus, the influence of object typicality on memory performance in VR environments requires further exploration.

Finally, the present study examined recognition confidence, finding this to be higher in the real-life condition than the HMD-VR and 2D picture conditions, with respect to both correct (i.e., hits, correct rejections) and incorrect responses (i.e., misses, false alarms). These results align with those of Mania et al. (2003), who compared participants in three different conditions (i.e., a real room, an HMD virtual room, a desktop display of a room) with respect to accuracy and confidence, finding that participants in the real room condition had the most accurate and confident memory performance, whereas participants in the HMD virtual room were slightly less accurate and confident, and participants who were exposed to the desktop display of the room were the least accurate and confident.

Overall, it is worth mentioning that all the findings’ comparisons in VR research should be interpreted with caution, considering the different technical features of the systems and the methodological choices by researches, as highlighted by Smith (2019) in his review.

Before drawing conclusions, some limitations of the present study must be mentioned. First, to maintain stimuli similarity across the three conditions, the HMD-VR stimulus consisted of a panorama 360° picture of the academic office and not an ad hoc, 3D, VR-generated scenario. Therefore, the type of VR stimulus might have jeopardized memory performance in the HMD-VR coding condition, making it more comparable to the 2D picture coding condition than to the real-life coding condition. Indeed, Johnson-Glenberg et al. (2021) claimed that, for VR to be effective, it should preserve some critical characteristics, such as a feeling of presence and interactivity with the environment. As the present study aimed at investigating differences in memory performance related to the coding context and the influence of stimuli emotional features and typicality on memory performance, no other factors related to the VR environment were specifically examined. However, it should be noted that participants in the HMD-VR coding condition rated an appropriate sense of presence and that in all experimental conditions participants could not interact with the environment. Second, as demonstrated in the literature (Godden & Baddeley, 1975), memory performance is superior when information is recalled in the same context as that of its coding. Thus, as all groups performed the memory recognition task on a computer, it is possible that the performance of participants in the VR condition was further decreased by the different retrieval context that, instead, was the same coding environment for participants in the 2D picture condition. Lastly, in the VR coding context, participants had to wear a Meta Quest 2 device, with which most of the sample reported no prior experience. Thus, although participants were advised during the training phase to wear the device and adjust it for comfort, the device may have still affected participants’ attention during the coding process.

Taken together, the results of the present study highlight that, although VR may contribute to the development of more ecologically valid experimental settings, to date, memory performance in VR settings cannot be considered fully comparable to that of real-life contexts, in which both recall, and recognition are superior. Nevertheless, it is important that future studies take into account also the differences in memory processes when examining memory performance in VR compared to in-vivo and 2D conditions. Indeed, previous studies suggested that VR and 2D conditions differ in the electrophysiological correlates elicited (Kisker et al., 2021a, 2021b). Furthermore, in the present study, an interesting effect of emotional valence emerged in the VR condition, particularly for objects with positive valence. This finding supports previous research underlining the crucial role of emotional valance on memory performance, and it opens up for future research on this topic in the field of VR.

In conclusion, further research is needed to create VR environments that are sufficiently comparable to real-life contexts to obtain reliable measures of cognitive performance. Importantly, such research should also consider critical characteristics of VR, including interactivity.

### Supplementary Information

Below is the link to the electronic supplementary material.

Supplementary file1 (DOCX 1497 KB)

## Data Availability

The datasets generated during the current study are available from the corresponding author on reasonable request. The experiment was not preregistered.

## Footnotes

1 The Meta Quest 2 is a standalone head-mounted display (HMD) with a 72 Hz LCD screen and a resolution of 1832 × 1920 pixels per eye. The device is placed in front of the eyes and covers the entire field of vision. It also includes two hand-held controllers that simulate hands. In the present study, only one controller was used by participants to virtually enter the room.

2 In a further set of exploratory analyses, RBMT and CBT scores were entered as covariates in all ANCOVAs. Neither of these variables emerged as significant covariates. Therefore, the more parsimonious models were reported.
