# Supplementary material for: Influence of stimuli emotional features and typicality on memory performance: insights from a virtual reality context
Source: Psychol Res. 2023 Jun 27;88(1):257–70. doi: 10.1007/s00426-023-01850-8 (PMC10805939; doi:10.1007/s00426-023-01850-8)
Supplement: Supplementary file 1 — Supplementary file1 (DOCX 1497 KB) [file 426_2023_1850_MOESM1_ESM.docx]

*To Psychological Research*

**Influence of Stimuli Emotional Features and Typicality on Memory Performance: Insights from a Virtual Reality Context**

Irene Ceccato*^1^, Eleonora Ricci*^1^, Cristina Mazza^1^, Emanuela Bartolini^1^, Adolfo Di Crosta^2^,
Pasquale La Malva^2^, Silvia Biondi^3^, Marco Colasanti^2^, Nicola Mammarella^2^,

Rocco Palumbo^2§^, Paolo Roma^3^, Alberto Di Domenico^2^

**^§^Corresponding Author**

Prof. Rocco Palumbo, Department of Psychological, Health and Territorial Sciences, University "G. d'Annunzio" of Chieti-Pescara, Via dei Vestini 31, 66100, Chieti, Italy.

E-mail: [rocco.palumbo@unich.it](mailto:rocco.palumbo@unich.it)

**Supplementary Information**

**Rating study**

An independent sample of young adults (N = 138; age: *M* = 24.08, *SD* = 3.22; 90% women) was recruited to rate the objects present in the room. Participants were mostly students (95%) and participated as volunteers. Written informed consent was obtained. The study was administered online via Qualtrics. Participants were requested to watch the seven pictures of the room shown to participants in the 2D pictures group and had to indicate in which pictures (one or more) a given object was visible. Participants can visually explore the seven pictures with no time limit. No feedback was provided on the accuracy of the answer. For each object, after the identification phase, participants were requested to look at a single picture selected among the seven previously shown, in which the object was highlighted (figure S1), and to rate it on three dimensions: valence, arousal, and typicity. A 9-point Likert scale was used for the three dimensions investigated. For valence, the scale ranged from *Negative* to *Positive*; for arousal, the scale ranged from *Calm* to *Excited*; for typicality, the scale ranged from *Not at all typical* to *Very typical*. Based on the results, for each dimension separately, objects were coded as “Low” when the average score was lower than 5 out of 9 and “High” when the average score was equal to or greater than 5. Table S1 reports the average scores and the categorizations of each object in the three dimensions.

**Table S1.** *Objects’ rating and classification on the three dimensions rated.*

|  |  | **Valence** | | **Arousal** | | **Typicity** | |
| --- | --- | --- | --- | --- | --- | --- | --- |
| **code** | **description** | **Mean** | **Category** | **Mean** | **Category** | **Mean** | **Category** |
| 1 | Crushed paper | 3.54 | LOW | 5.64 | HIGH | 6.41 | HIGH |
| 2 | Dirty tissue | 3.37 | LOW | 5.33 | HIGH | 5.12 | HIGH |
| 3 | Broken pencil | 2.98 | LOW | 6.30 | HIGH | 5.06 | HIGH |
| 4 | Ripped book | 3.02 | LOW | 6.15 | HIGH | 5.00 | HIGH |
| 5 | Dying plant | 3.01 | LOW | 4.72 | LOW | 3.27 | LOW |
| 6 | Full ashtray | 2.26 | LOW | 6.01 | HIGH | 3.46 | LOW |
| 7 | Dirty-water bottle | 2.22 | LOW | 5.77 | HIGH | 1.81 | LOW |
| 8 | Broken chair | 2.15 | LOW | 6.33 | HIGH | 3.61 | LOW |
| 9 | Tossed surgical mask | 3.04 | LOW | 5.78 | HIGH | 3.93 | LOW |
| 10 | Spilling waste basket | 3.40 | LOW | 5.25 | HIGH | 7.26 | HIGH |
| 11 | Terroristic attack painting | 2.28 | LOW | 7.15 | HIGH | 1.93 | LOW |
| 12 | Book | 7.14 | HIGH | 3.54 | LOW | 8.48 | HIGH |
| 13 | Coffe maker | 7.25 | HIGH | 3.95 | LOW | 6.47 | HIGH |
| 14 | Pen | 6.80 | HIGH | 3.54 | LOW | 8.69 | HIGH |
| 15 | Chair | 6.72 | HIGH | 3.09 | LOW | 8.50 | HIGH |
| 16 | Mini cooler | 6.64 | HIGH | 3.59 | LOW | 3.78 | LOW |
| 17 | Tissue box | 5.62 | HIGH | 4.09 | LOW | 6.17 | HIGH |
| 18 | Computer and mouse | 6.97 | HIGH | 3.70 | LOW | 8.22 | HIGH |
| 19 | Mug | 6.37 | HIGH | 3.15 | LOW | 5.55 | HIGH |
| 20 | Sunglasses | 6.09 | HIGH | 3.74 | LOW | 3.11 | LOW |
| 21 | Glass | 5.54 | HIGH | 4.09 | LOW | 6.19 | HIGH |
| 22 | City painting | 5.02 | HIGH | 5.07 | HIGH | 2.93 | LOW |
| 23 | Flowers in a vase | 7.12 | HIGH | 2.86 | LOW | 4.93 | LOW |
| 24 | Fruit juice | 5.92 | HIGH | 3.89 | LOW | 3.81 | LOW |
| 25 | Banknote | 6.41 | HIGH | 4.81 | LOW | 2.38 | LOW |
| 26 | Cookies’ box | 6.67 | HIGH | 3.53 | LOW | 3.94 | LOW |
| 27 | Candies in a bowl | 7.14 | HIGH | 3.30 | LOW | 5.17 | HIGH |
| 28 | Star-shaped paperweight | 6.28 | HIGH | 3.49 | LOW | 5.89 | HIGH |
| 29 | Dreamcatcher | 6.78 | HIGH | 3.01 | LOW | 2.51 | LOW |
| 30 | Trumpet | 5.49 | HIGH | 5.01 | HIGH | 2.09 | LOW |
| 31 | Rubik’s cube | 5.93 | HIGH | 4.71 | LOW | 3.16 | LOW |
| 32 | Bonbonnière | 7.22 | HIGH | 3.92 | LOW | 4.12 | LOW |
| 33 | Island picture | 7.42 | HIGH | 2.18 | LOW | 3.85 | LOW |
| 34 | Closet | 5.73 | HIGH | 3.93 | LOW | 5.38 | HIGH |
| 35 | Furniture with shelves | 5.54 | HIGH | 4.09 | LOW | 6.35 | HIGH |
| 36 | Window | 6.12 | HIGH | 3.61 | LOW | 7.43 | HIGH |
| 37 | Air conditioner | 7.11 | HIGH | 3.17 | LOW | 6.70 | HIGH |
| 38 | Shelves | 6.20 | HIGH | 3.59 | LOW | 7.17 | HIGH |
| 39 | Radiator | 7.27 | HIGH | 2.68 | LOW | 7.74 | HIGH |
| 40 | Printer | 6.63 | HIGH | 3.52 | LOW | 7.93 | HIGH |
| 41 | Dresser | 6.23 | HIGH | 3.55 | LOW | 7.59 | HIGH |
| 42 | Whiteboard | 6.31 | HIGH | 4.31 | LOW | 6.74 | HIGH |
| 43 | Telephone | 6.48 | HIGH | 4.32 | LOW | 6.99 | HIGH |
| 44 | Desk | 6.80 | HIGH | 3.51 | LOW | 8.42 | HIGH |
| 45 | Calendar | 6.11 | HIGH | 4.16 | LOW | 7.55 | HIGH |
| 46 | Coat hanger | 6.20 | HIGH | 3.49 | LOW | 6.80 | HIGH |
| 47 | Two-door furniture | 5.50 | HIGH | 3.91 | LOW | 6.06 | HIGH |
| 48 | Door | 6.48 | HIGH | 3.97 | LOW | 8.31 | HIGH |
| 49 | Emergency light | 5.82 | HIGH | 4.60 | LOW | 6.42 | HIGH |
| 50 | Socket | 6.09 | HIGH | 4.18 | LOW | 8.15 | HIGH |
| 51 | Writing “The Boss” | 4.42 | LOW | 5.05 | HIGH | 2.07 | LOW |
| 52 | Succulent plant | 6.02 | HIGH | 3.42 | LOW | 5.30 | HIGH |
| 53 | Graduation certificates (on the wall) | 7.57 | HIGH | 3.99 | LOW | 7.36 | HIGH |

**Fig.S1** *Example of the picture shown during the rating phase. The target object (crushed paper) was highlighted with a red circle.*


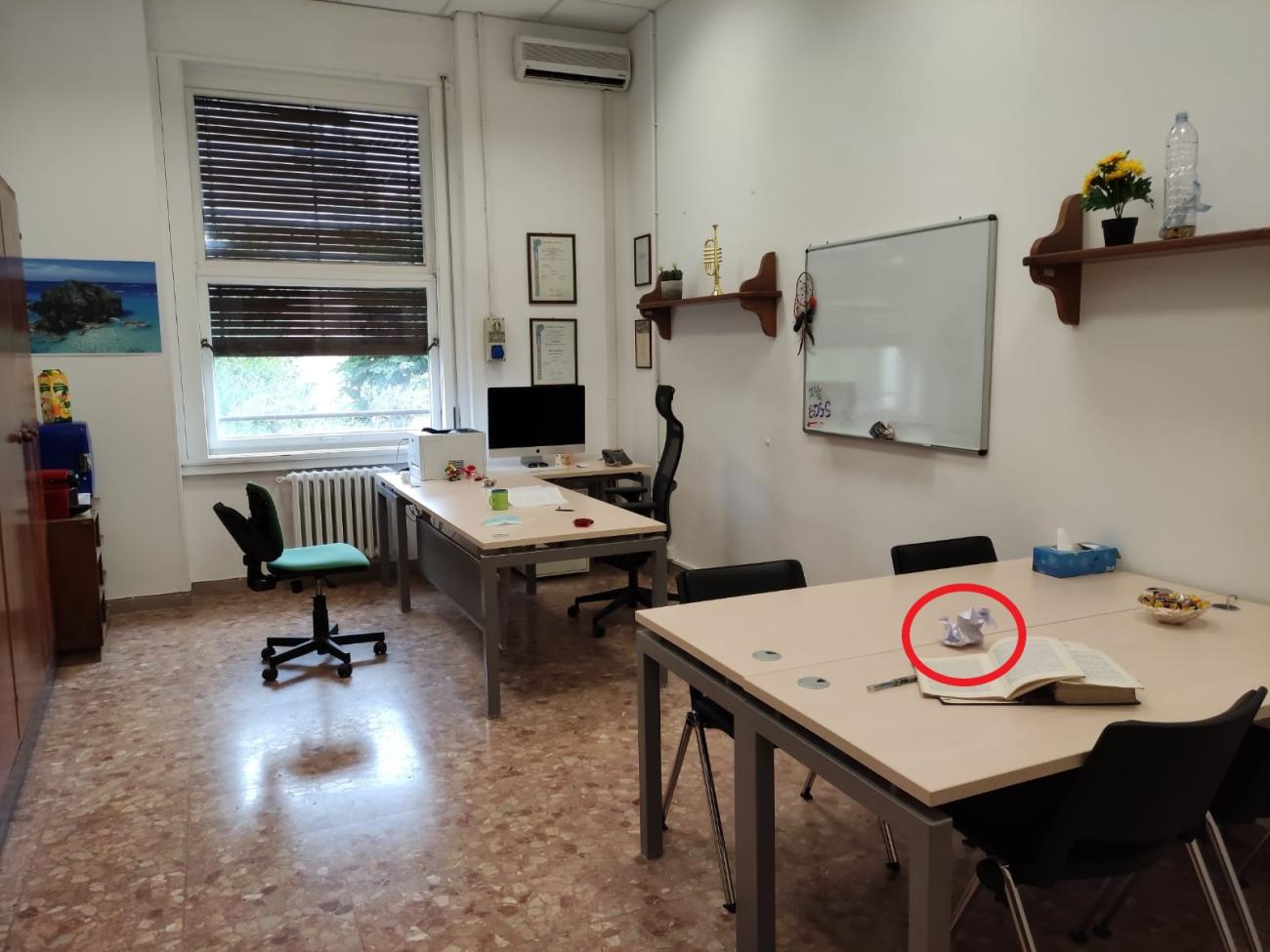


**Fig.S2** *Pictures used in the 2D condition.*


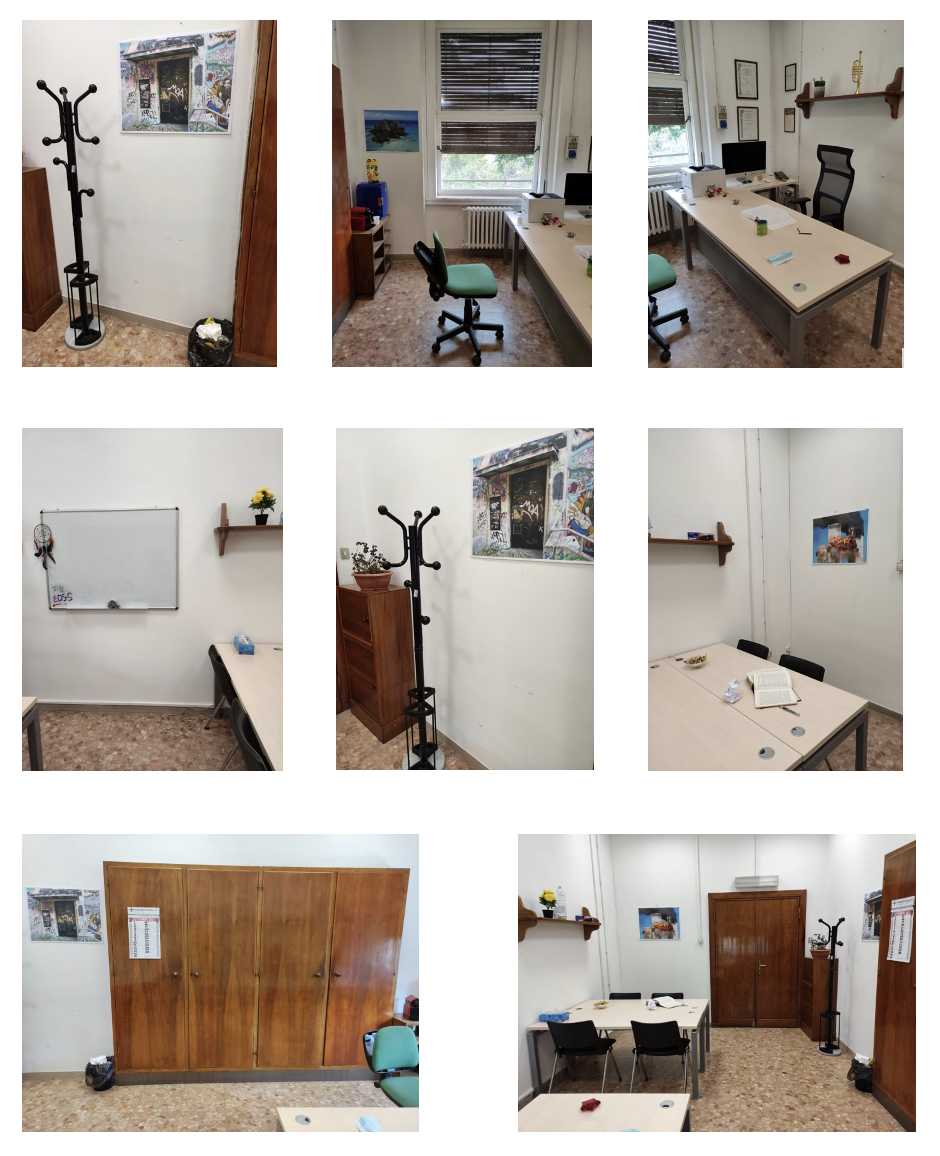


*Note. The last picture is also included in the manuscript (Fig.2).*
